# Epidemiological profile of neuroendocrine tumors in adults in Brazil

**DOI:** 10.20945/2359-4292-2023-0013

**Published:** 2024-05-07

**Authors:** Priscilla Brunelli Pujatti, Verônica Paim, Rinaldo Gonçalves, Bruno Vilhena, Anke Bergmann, Luiz Cláudio Santos Thuler, Daniel Bulzico

**Affiliations:** 1 Instituto Nacional de Câncer Serviço de Medicina Nuclear Rio de Janeiro RJ Brasil Serviço de Medicina Nuclear, Instituto Nacional de Câncer (INCA), Rio de Janeiro, RJ, Brasil; 2 Instituto Nacional de Câncer Seção de Cirurgia Abdominopélvica Rio de Janeiro RJ Brasil Seção de Cirurgia Abdominopélvica, Instituto Nacional de Câncer (INCA), Rio de Janeiro, RJ, Brasil; 3 Instituto Nacional de Câncer Seção de Oncologia Clínica Rio de Janeiro RJ Brasil Seção de Oncologia Clínica, Instituto Nacional de Câncer (INCA), Rio de Janeiro, RJ, Brasil; 4 Instituto Nacional de Câncer Rio de Janeiro RJ Brasil Programa de Epidemiologia Clínica, Instituto Nacional de Câncer (INCA), Rio de Janeiro, RJ, Brasil; 5 Instituto Nacional de Câncer Unidade de Oncologia Endócrina Rio de Janeiro RJ Brasil Unidade de Oncologia Endócrina, Instituto Nacional de Câncer (INCA), Rio de Janeiro, RJ, Brasil

**Keywords:** Neuroendocrine tumor, diagnosis, survival, epidemiology

## Abstract

**Objective:**

Neuroendocrine tumors (NETs) are a set of diseases that originate from neuroendocrine cells, which comprises a diffuse endocrine system present in various organs of the body. These tumors are more frequent in the gastrointestinal tract (70%) and the bronchopulmonary system (20%-30%). A NET incidence rate of 1-5 per 100,000 inhabitants has been estimated for several European countries and the USA employing 20 years of data. However, no comprehensive studies on this rare neoplasm are available in Brazil. In this context, the aim of this study was to characterize the epidemiological NET profile in the country.

**Material and methods:**

This is a retrospective descriptive observational study based on data from Hospital Cancer Records available at the Brazilian National Cancer Institute and the São Paulo Oncocentro Foundation. Demographic, clinical and treatment-related variables were analyzed from selected cases employing descriptive statistics.

**Results and Conclusion:**

A total of 15,859 cases were identified, most occurring in males (53.4%) and in individuals under 65 years old (63.3%). Small cell carcinoma was the most frequent histological type (46.7%). Bronchopulmonary tumors were the most frequent NETs, followed by pancreatic tumors, with cases mostly concentrated in high complexity centers in the Brazilian Southeast and treated mainly with surgery and chemotherapy, with over half of the patients diagnosed in advanced stages of the disease.

## INTRODUCTION

Neuroendocrine tumors (NETs) are a set of diseases that originate from neuroendocrine cells, which consists a diffuse endocrine system present in various organs of the body (1). These tumors are characterized by their ability to synthesize and secrete monoamines (2) and are more frequent in the gastrointestinal tract (70%) and the bronchopulmonary system (20%-30%), although they may rarely affect other sites, such as the skin, head, neck and genitourinary system, comprising less than 10% of all cases (3). Neuroendocrine tumors are classified according to their differentiation and proliferation rates, with grade 1 and grade 2 cases comprising well-differentiated tumors, differentiated by their proliferation index, where grade 1 tumors present a ki-67 index less than or equal to 3%, grade 2 tumors, ranging from 3 to 20%, and grade 3 comprising poorly differentiated tumors (4).

The most prevalent symptoms in gastrointestinal origin NETs are diarrhea and abdominal pain, which are common to several diseases, which makes NET diagnosis difficult in early stages (5,6). The most traditional immunohistochemical markers employed for diagnosis are synaptophysin and chromogranin A (7). Primary tumor surgical removal is performed as a first-line treatment, while in case of metastasis, somatostatin analogues are used (5). Treatment update has been observed over time, with the introduction of new drugs, such as the mammalian target protein inhibitor of rapamycin (mTOR), Sunitinib, Everolimus and radionuclide therapy employing [^177^Lu]Lu-DOTATATE (8).

A NET incidence rate of 1-5 per 100,000 inhabitants has been estimated for USA and several European countries employing 20 years of data, confirming that NETs are a rare neoplasm (2). In other parts of the world, such as Taiwan, cancer registries have indicated increasing numbers of cases over time, from 0.244 to 3.162 per 100,000 population between 1996 and 2015 (2). In Brazil, two articles in this field have been published, *i.e.*, Younes, who employed a database from 32 centers, and Silveira et al., who assessed a single center data. Therefore, no comprehensive studies are available in Brazil to date, even when considering national registries (3,9).

Considering the lack of NET data in Brazil, its rareness and diagnosis difficulty, the aim of the present study was to characterize epidemiological profile of NETs in adults in Brazil.

## MATERIALS AND METHODS

This is a retrospective descriptive observational assessment based on secondary data from Hospital Cancer Registries (HCR) available at Brazilian National Cancer Institute (*Instituto Nacional de Câncer* – INCA) and São Paulo Oncocentro Foundation (*Fundação Oncocentro de São Paulo* – FOSP). HCR are centers for the collection, storage, processing, and systematic and continuous analysis of information from patients with a confirmed cancer diagnosis treated in a hospital unit. The information produced in HCR allows for the monitoring of patient care. Its main function is clinical, serving as a resource to track and evaluate the quality of work performed in hospitals, including outcomes of cancer treatment. To consolidate the information, most HCRs use the SisHCR, a data computerization system developed and provided by INCA. The databases, consolidated according to the year of the first consultation at the reporting hospital, are sent to compose the national database of hospital cancer records, under the custody of INCA, through the IntegradorRHC (HCR Integrator). The operation of an HCR and the regular submission of data to the IntegradorRHC are mandatory for hospitals accredited in Specialized Oncology Care under the Brazilian Unified Health System (SUS) and optional for non-accredited hospitals.

Analytical cases concerning patients diagnosed from 2000 to 2019 were analyzed. As no specific NET topography and histological type coding is available, cases presenting the following topographies (ICD) were included: C16 (stomach cancer); C17 (small intestine); C18 (colon); C19 (rectosigmoid junction); C20 (rectum); C25 (pancreas); C26 (other digestive organs and poorly defined locations in the digestive tract); C34 (bronchi and lungs) and C75 (other endocrine glands and related structures); and which presented, concurrently, the following histological types: 8013/3 (Large cell neuroendocrine carcinoma); 8041/3 (Small cell carcinoma); 8150/3 (Islet cell carcinoma); 8150/1 (Islet Cell Tumor, NOS); 8151/3 (Malignant Insulinoma); 8152/1 (Glucagonoma, NOS); 8152/3 (Malignant Glucagonoma); 8153/1 (Gastrinoma, NOS); 8153/3 (Malignant gastrinoma); 8155/3 (Malignant Vipoma); 8156/1 (Somatostinoma, NOS); 8156/3 (Malignant somatostinoma); 8240/1 (Carcinoid tumor of uncertain malignancy); 8240/3 (NOS carcinoid tumor); 8241/3 (Enterochromaffin cell carcinoid); 8242/1 (Enterochromaffin-like cell carcinoid, NOS); 8242/3 (Malignant Enterochromaffin-like Cell Tumor); 8243/3 (Goblet cell carcinoid); 8244/3 (Compound carcinoid); 8245/1 (Tubular Carcinoid); 8245/3 (Adenocarcinoid tumor); 8246/3 (Neuroendocrine Carcinoma, NOS); 8248/1 (Apudoma) and 8249/3 (Atypical carcinoid tumor) (10).

Patients aged > 90 (19 cases) and < 18 (130 cases) and cases in which treatment began prior to the diagnosis (negative time, 609 cases) were excluded.

Demographic, clinical and treatment-related variables were analyzed. Demographic variables of "no information" in over 50% of the cases were excluded from the analysis.

A descriptive analysis concerning study population characteristics was performed through tendency and dispersion measures for continuous variables and absolute and relative frequencies for categorical variables. The statistical program SPSS v. 17.0 was used.

This study was approved by Research Ethics Committee (CAAE 0104.0.007.000-11). In addition, assessment to use secondary Internet based public data is waived as disposed by Brazilian National Health Council (*Conselho Nacional de Saúde*) Resolutions no. 196/1996, 466/2012 and 510/2016.

## RESULTS

Considering the inclusion criteria, a total of 15,859 NET cases were identified in Brazil during the studied period, 53.4% in males and 63.3% in individuals younger than 65 years old. Most of cases were identified in the Southeast region of the country. Other demographic characteristics of the analyzed cases are displayed in Table 1.

**Table 1:** Demographic characteristics of neuroendocrine tumor cases reported in Brazil between 2000 and 2019 (n = 15,859)

Demographic Characteristic		n	%
Sex			
	Male	8,462	53.4
	Female	7,397	46.6
Age (years)			
	18 to 65	10,035	63.3
	>65	5,824	36.7
Schooling			
	<8 years	5,979	37.7
	>8 years	5,674	35.8
	No information	4,206	26.5
Birth region			
	Southeast	7,744	48.9
	South	3,257	20.6
	North East	3,124	19.6
	North	351	2.2
	Midwest	305	2.0
	No information	1,078	6.9
Residence region			
	Southeast	9,447	59.6
	South	3,303	20.8
	North East	2,067	13.2
	North	520	3.3
	Midwest	465	2.9
	No information	57	0.4

The most prevalent NET topography were bronchi and lungs (66.5%), followed by pancreas (9.4%) and gastrointestinal tract – stomach (7.4%), small intestine (6.1%), colon (5.9%) and rectal (3.9%). The most frequent histological types were small cell carcinoma (46.7%), neuroendocrine carcinoma, Not otherwise specified (NOS) (30.8%) and NOS carcinoid tumor (14.5 %). Those three histological types were responsible for 92 % of total cases. Also, most cases comprised stage IV tumors (57.7%) (Table 2).

**Table 2:** Clinical characteristics of neuroendocrine tumor cases in Brazil between 2000 and 2019 (n = 15,859)

Characteristic		n	%
Topography (CID)			
	Bronchi and lungs	10,544	66.5
	Pancreas	1,494	9.4
	Stomach	1,175	7.4
	Small intestine	974	6.1
	Colon	941	5.9
	Rectal	617	3.9
	Other digestive organs and poorly defined locations in the digestive tract	43	0.3
	Rectosigmoid junction	42	0.3
	Endocrine glands and related structures	11	0.1
Histological type			
	Small cell carcinoma	7,400	46.7
	Neuroendocrine Carcinoma, NOS	4,881	30.8
	NOS carcinoid tumor	2,293	14.5
	Large cell neuroendocrine carcinoma	506	3.2
	Atypical carcinoid tumor	299	1.9
	Carcinoid tumor of uncertain malignancy	170	1.1
	Islet cell carcinoma	65	0.4
	Compound carcinoid	63	0.4
	Malignant insulinoma	41	0.3
	Goblet cell carcinoid	32	0.2
	Adenocarcinoid tumor	29	0.2
	Enterochromaffin cell carcinoid	19	0.1
	Tubular carcinoid	13	0.1
	Islet cell tumor, NOS	13	0.1
	malignant gastrinoma	10	0.1
	Gastronoma, NOS	8	0.1
	Enterochromaffin-like cell carcinoid, NOS	5	0.0
	Malignant enterochromaffin-like cell tumor	5	0.0
	Malignant Glucagonoma	3	0.0
	Apudoma	1	0.0
	Glucagonoma, NOS	1	0.0
	Somatostinoma, NOS	1	0.0
	Malignant vipoma	1	0.0
Clinical staging			
	0	20	0.1
	I	1,227	7.7
	II	685	4.31
	III	2,258	14.3
	IV	5,724	36.1
	No information	5,945	37.5

Legend: NOS – not otherwise specified.

The main therapeutic modality for treated patients was chemotherapy, followed by surgery and combined radiotherapy and chemotherapy (27.4%, 25.6% and 16.9%, respectively). Information of first follow up was available for only 7683 patients (48.44%). Among them, 24.4% died, 17.4% had stable disease, 15.7% had complete or partial remission and 15.7% had disease progression (Table 3).

**Table 3:** Neuroendocrine tumor treatments in Brazil between 2000 and 2019 (n = 15,859)

Characteristic		n	%
Treatment (n = 15,069)			
	Chemotherapy	4,343	27.4
	Surgery	4,057	25.6
	Radiotherapy	733	4.6
	Radiotherapy + Chemotherapy	2,674	16.9
	Surgery + Chemotherapy	944	6.0
	Surgery + Radiotherapy	118	0.7
	Surgery + Chemotherapy + Radiotherapy	330	2.1
	No treatment	1,802	11.4
	No information	68	0.4
Disease status at the end of primary treatment (n = 7,683)			
	Complete remission	818	10.6
	Partial remission	389	5.1
	Stable disease	1,334	17.4
	Disease in progress	1204	15.7
	Oncological therapeutic support	260	3.4
	Death	1,871	24.4
	No information	1,807	23.5

Considering the healthcare unit from where patients were registered, most cases were treated in specialized units (53.3%) from the Southeast Brazil (62.2%) (Table 4).

**Table 4:** Characteristics of the neuroendocrine tumor case treatments units in Brazil between 2000 and 2019

Characteristic		n	%
Reference Cancer Center or Unit			
	Yes	8,433	53.3
	No	7,388	46.7
Hospital Unit Region			
	Southeast	9,875	62.2
	South	3,311	20.9
	Northeast	2,002	12.4
	North	370	2.4
	Midwest	301	1.9

## DISCUSSION

The present study comprises a real-world assessment of NET cases in Brazil from 2000 and 2019, the largest historical survey of this kind conducted in the country. Most identified cases were registered in high-complexity cancer centers located mainly in the Brazilian Southeast, and over half of the patients were diagnosed in advanced stages.

Epidemiological studies carried out in the last decade describe a progressive increase in the incidence and especially in the prevalence of NET cases in different parts of the world (11-13), although the causes for this increase are not yet fully known. One of the main obstacles concerning epidemiological NET surveys is the absence of a specific coding in the International Code of Diseases (ICD-10). Consequently, misclassifications are frequent, compromising data reliability. Herein, a strategy of cross-checking cases registered as ICD-10, commonly employed for NET classification in different primary sites, was employed, concerning cases recorded as malignant neoplasms of neuroendocrine origin according to the Oncology ICD (ICD-O). Thus, we believe that the chance of losing cases was reduced to the lowest possible. Herein, bronchopulmonary NET cases were the most frequent, corroborating other international series (11,14). Pancreas tumors were the most frequent among extrapulmonary NET cases. The order of primary location pancreas and gastrointestinal tract NET frequencies varies in different centers worldwide, probably due to local issues, such as general population cancer screening policies (15). However, local aspects such as lifestyle habits, environmental exposure, and genetic population patterns cannot be excluded.

Regarding demographic data, most cases were detected in men, similarly to that reported in a NET epidemiological study carried out in Japan (14). However, data indicate a predominance of cases in women in the US (11). Regarding registration, almost half of the cases were registered in Brazilian Southeast, the most populous region in the country. However, other issues such as the greater concentration of high complexity centers and greater access to specialized health services in the states located in this region may be associated with a greater probability of the diagnosis of rare diseases such as NET. However, it is important to note that a total of 46% of the cases were treated in less complex oncology centers (CACON/UNACON).

The elusive character of the disease, as well as the lack of knowledge among both patients and healthcare professionals regarding rare diseases, leads to a significant proportion of NET cases being diagnosed at advanced stages, often with distant metastasis (11-13). Although a 30.8% frequency of neuroendocrine carcinomas was detected in the histological type analysis, histological data regarding tumor morphology, mitotic index and Ki-67 expression were not available in the assessed registry bases, hindering tumor confirmation and reliable analyses when comparing clinical outcomes between well- and poorly differentiated tumors. Furthermore, clinical data are not included in the assessed registries, so information on clinical hormonal hypersecretion syndrome in cases of pancreatic NET and carcinoid syndrome were not available. Furthermore, histological diagnoses could not be reviewed in light of the current recommendation for NET classification according to the World Health Organization (WHO) (10).

Surgery and chemotherapy were the most applied initial treatments, as expected. Although somatostatin analogues are essential in NET treatment, the percentage of patients treated with these compounds could not be determined. However, this treatment may have been included as an option at the assessed treatment centers within the chemotherapy category.

While this study marks a significant milestone in understanding NET patterns and treatment in Brazil, it’s important to note that the data presented here may not comprise all NET cases in the country during the study period, as cases treated at medical centers not connected to the HCR or FOSP databases were not considered. Thus, NET incidence and prevalence rates in Brazil were not calculated, as they did not fit the study scope and would not be accurately determined.

Limitations in this study are inherent to its nature, involving an analysis based on large-scale data records from general databases where data quality cannot be controlled. We were unable to access individual clinical, histological, and Ki-67 immunoexpression data, which in turn hindered the classification of Neuroendocrine Tumors (NETs) based on differentiation degree, thereby limiting a more in-depth analysis of the clinical behavior of various NET types. Additionally, information regarding subsequent therapies after initial treatment was not accessible for analysis and follow up was not available for all patients. In conclusion, this study presents an original and robust real-life NET survey concerning cases in Brazil over a period of 20 years. Bronchopulmonary NET cases were the most prevalent, followed by pancreatic NETs, concentrated in high-complexity centers in Southeast Brazil and treated mainly with surgery and chemotherapy. We emphasize the need for this data to be refined for a more detailed analysis of the current patient NET care state in Brazil.
